# Supplementary material for: Middle East Respiratory Syndrome Coronavirus (MERS-CoV) Seropositive Camel Handlers in Kenya
Source: Viruses. 2020 Apr 3;12(4):396. doi: 10.3390/v12040396 (PMC7232417; doi:10.3390/v12040396)
Supplement: Supplementary file 1 [file viruses-12-00396-s001.pdf]

Supplementary Materials

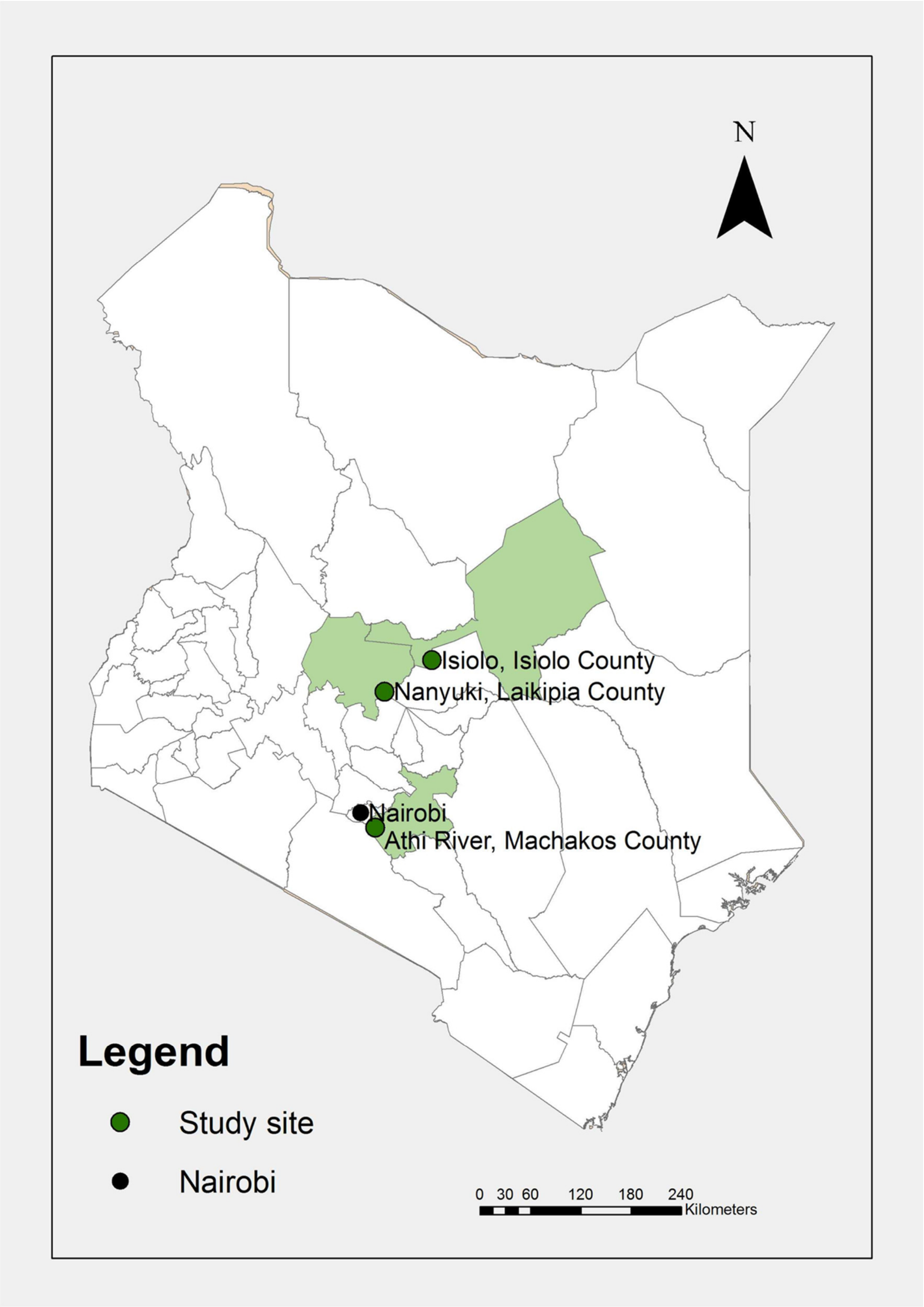

Figure S1 Map of sites where camel handlers were sampled in Kenya in 2016. Counties are indicated in light green and the locations of the slaughterhouses in dark green

Table S1 Results of testing selected sera from slaughterhouse workers and camel herders for antibodies to MERS-CoV by Euroimmun ELISA, S1 ELISA and PRNT. Positive results are shaded.

ID	Classification	Euroimmun extinction ratio	S1 ELISA OD	$PRNT_{50}$
UZ035342	Herder	0.20	0.20	<20
UZ035755		0.50	0.44	<20
UZ035881		0.22	0.23	<20
UZ036131		0.41	0.43	<20
UZ036167		0.59	0.69	<20
UZ036168		0.06	0.29	<20
UZ036195		0.07	0.39	<20
UZ035771	Worker	0.38	0.29	<20
UZ035899		0.20	0.26	<20
UZ035901		0.15	0.42	<20
UZ035902		0.17	0.31	<20
UZ035903		0.14	0.67	40
UZ035906		0.52	0.42	<20
UZ035917		0.45	0.23	<20

UZ035924		0.37	0.61	20
UZ035926		0.11	0.31	<20
UZ035929		0.14	0.57	20
UZ035981		0.09	0.26	<20
UZ035988		0.28	0.27	<20
UZ035994		0.11	0.17	<20
UZ035998		0.31	0.62	40

Table S2 Demographics and practices of camel slaughterhouse workers in three counties, Machakos, Laikipia and Isiolo, Kenya, in 2016

Variable	Machakos n=5	Laikipia n=16	Isiolo n=37	Total n=58
**Gender**				
Male	5	16	26	47
Female	0	0	11	11
**Age**				
<20	1	0	1	2
20-39	2	11	24	37
40-59	2	5	10	17
>60	0	0	2	2
**Job**				
Slaughterman	0	2	8	10

Flayer	1	5	7	13
Cleaner	1	5	5	11
Other	3	4	17	24
**Drink Camel Milk**				
No	3	11	15	29
Boiled	0	3	5	8
Raw	2	2	17	21
**Milk camel**				
No	4	14	23	41
Yes	1	2	14	17
**Drink blood**				
No	5	16	31	52
Yes	0	0	6	6

Table S3 Univariable risk factor analysis for MERS-CoV seropositivity in slaughterhouse workers at Isiolo Camel Slaughterhouse, Kenya in 2016

Variable	Isiolo n=37 (%)	Odds Ratio (95% CI)	p-value
**Gender**			
Male	3/26 (11.5)	1.26 (0.09-72.58)	1
Female	1/11 (9.1)	1	

**Age**			
<40	2/25 (8.0)	1	
40 and over	2/12 (16.7)	2.04 (0.13-31.43)	0.60
**Job**			
Slaughterman	3/8 (37.5)	10.09 (0.70-587.39)	0.50
Other	1/29 (3.4)	1	
**Drink Camel Milk**			
No	1/15 (6.7)	1	
Yes	3/22 (13.6)	2.01 (0.15-114.36)	0.64
**Milk camel**			
No	1/23 (4.3)	1	
Yes	3/14 (21.4)	4.74 (0.34-269.27)	0.29
**Drink blood**			
No	1/31 (3.2)	1	
Yes	3/6 (50.0)	14.02 (0.95-833.42)	0.03
